# AI-assisted multimodal data integration for precision oncology

**DOI:** 10.3389/frai.2026.1917442

**Published:** 2026-09-07

**Authors:** Alexandra Chera, Ana-Maria Chirilaș, Amada El-Sabeh, Mircea Crețu Stancu, Octavian Bucur

**Affiliations:** 1 Genomics Research and Development Institute, Bucharest, Romania; 2 Carol Davila University of Medicine and Pharmacy, Bucharest, Romania; 3 Viron Molecular Medicine Institute, Boston, MA, United States

**Keywords:** artificial intelligence (AI), deep learning, multimodal data integration, multi-omics, precision oncology

## Abstract

The complexity of data pertaining to patients diagnosed with cancer requires a shift from fragmented, unimodal diagnostics towards multimodal artificial intelligence (MAI) in order to achieve true precision oncology. In this literature review we examined the landscape of unimodal data modalities used in oncological practice including clinical records, multi-scale imaging (radiology and histopathology), and multi-omics signatures, alongside a critical comparison of the deep-learning architectures and integrative frameworks used to combine these data sources into advanced predictive models. By using fusion strategies (early, late, intermediate, and hybrid), MAI models are able to bridge the gap between genotype and phenotype, uncovering biological interactions that remain invisible to single-modality analysis. Current applications demonstrate significant improvements in diagnostic sensitivity, automated tumor grading, and the prediction of complex clinical outcomes, such as immunotherapy response and overall survival, referencing leading-edge tools and frameworks currently used or in active research. However, the transition from research to clinical practice is hindered by limitations such as data fragmentation, demographic biases, limited model explainability, and evolving regulatory requirements. We further outline emerging directions, including multimodal foundation models, large language models, and retrieval-augmented, agent-based systems. We concluded that the convergence of multimodal data streams and biologically informed AI represents the essential step for the next generation of personalized cancer care.

## 1 Introduction

The integration of deep learning and big data is transforming numerous fields, particularly oncology (Al-Zoghby et al., 2025). Since complex molecular networks are involved in the physiopathology of cancer, there is an increasing need of dynamically addressing the biological information in a comprehensive manner (Wekesa and Kimwele, 2023). Conventional diagnostic workflows often rely on fragmented, unimodal data, leading to significant clinical vulnerabilities. A nationwide survey from 2024 conducted in Netherlands revealed that 32.1% of patients with rare cancers received an initial incorrect diagnosis, with 44.6% of these individuals subsequently receiving inappropriate treatments (Padilla et al., 2024). Time-to-diagnosis remains a critical bottleneck, averaging 13.5 weeks for lung cancer (Nalewaj et al., 2025) and 6.4 months for early-onset colorectal cancer (Demb et al., 2024). Such delays are fatal, considering every four-week delay in treatment increases mortality risk by 6–8% (Bowles, 2026), while a 12-week delay in breast cancer surgery increases mortality risk by 26% (Hanna et al., 2020). In lung cancer specifically, starting treatment more than 30 days post-diagnosis can reduce the median survival time from 14.8 months to 11 months (Nalewaj et al., 2025).

One of the culprits for delayed diagnosis is fragmented and, in many cases, unimodal data availability. Real-world studies of advanced non-small cell lung cancer (NSCLC) show that only 21% of patients had molecular testing results available at their initial oncology consultation, and in 19% of cases chemotherapy has to be initiated before obtaining molecular diagnoses, thus risking inappropriate treatment (Gregg et al., 2019). In the field of radiology, 72% of medical practitioners declare that they frequently require more clinical information than they receive for an accurate diagnosis, and 87% report that this additional data could modify their interpretive reports (Boonn and Langlotz, 2009).

To uncover the biological processes underlying neoplastic disease, it is essential to use a multidimensional approach, which involves analyzing multi-omics datasets with the aid of modern computational tools (Wekesa and Kimwele, 2023). A patient’s oncological state is characterized by a full spectrum of modalities, ranging from radiology and histology to genomics and electronic health records (EHR), with each providing vital additional insights (Stahlschmidt et al., 2022). Therefore, to reach a comprehensive picture of a patient’s condition, multimodal medicine must harmonize and integrate the numerous types of variables generated from each modality, the data (text, images, numerical) being classified as either continuous (quantitative) or categorical (qualitative). There can be mixed data types even within a single modality, for example in genomics, where continuous data describes DNA methylation (plus mRNA expression and protein concentration levels if extended to multi-omics), while categorical data is generated for mutation profiling (mutation types, pathogenicity, genomic alteration status) (Krones et al., 2025; Boie et al., 2026).

Artificial Intelligence (AI) is a broad field of computer science focused on developing systems capable of performing tasks that typically require human intelligence, such as complex decision-making and pattern recognition. AI encompasses specific computational subsets, including machine learning (ML) and deep learning (DL), which provide the mathematical and statistical foundations for these systems (Wekesa and Kimwele, 2023). ML enables algorithms to learn patterns and make predictions from vast datasets without being explicitly programmed. ML generally operates through three main paradigms: supervised learning using human-labeled data, unsupervised learning for clustering unlabeled instances, and reinforcement learning based on reward systems (Tomczak et al., 2015; Unger and Kather, 2024; Haq et al., 2025; Jandoubi and Akhloufi, 2025).

Traditionally, ML in healthcare, particularly in radiology, has focused on augmenting single-modality (unimodal) data analysis, which relies on a single data source, such as radiology images, clinical notes, or genomic profiles, to make diagnostic predictions. While this approach offers methodological simplicity and lower computational costs, it only provides a fragmented view and remains vulnerable to noise, errors, or missing data within that specific channel (Al-Zoghby et al., 2025). Hence, depending on a single data stream is not considered reliable enough in modern oncology (Haq et al., 2025), and despite the exponential growth of the high-dimensional healthcare data, until recently, many of the currently-used AI models still operated mainly in the realm of a single modality, unable to integrate the mixed data, thus diminishing their overall potential and neglecting the broader clinical context (Lipkova et al., 2022; Al-Zoghby et al., 2025).

Although unimodal AI has already started to improve outcomes, for example through increasing colonoscopy adenoma detection rates by up to 26% (Shiha et al., 2023), multimodal AI is essential to overcome data fragmentation. Multimodal integration enhances diagnostic precision by 5 to 15% over unimodal baselines (Jandoubi and Akhloufi, 2025) and reduces administrative burdens, studies showing that it could potentially save 145 working days in imaging workflows over 5 years (Dellamonica et al., 2025), thus facilitating true precision oncology. Because intricate disease processes cannot be fully understood when these diverse data sources are examined separately, utilizing a multimodal method that processes several data types has been proven to be essential (Jiao et al., 2024; Al-Zoghby et al., 2025). The comparative merits of these fusion strategies are examined critically in Section 5.1.

DL is a specialized advancement within ML that employs multi-layered artificial neural networks to automatically extract hierarchical, high-level features directly from raw, complex data like medical images and genomics (Al-Zoghby et al., 2025; Guarrasi et al., 2025). Unlike ML, which often requires manual feature engineering, DL excels at modeling non-linear relationships within high-dimensional heterogeneous data, making it the dominant approach for biomarker discovery and multimodal integration (Lipkova et al., 2022). Building on these capabilities, Multimodal Artificial Intelligence (MMAI) has emerged as a highly promising tool that integrates clinical and paraclinical data, cancer multi-omics and imaging data (histopathology, radiology), allowing DL models to connect diverse biological levels and enhance diagnostic precision (Jandoubi and Akhloufi, 2025; Dellamonica et al., 2025).

While traditional workflows still rely on manual reviews by pathologists and engineered computational pipelines, modern ML and DL tools offer new efficient ways to extract actionable insights from raw data sources (Unger and Kather, 2024). To simulate the clinical reasoning of human doctors, future computer-assisted diagnostic systems must process diverse data types simultaneously (Haq et al., 2025). Multimodal deep learning has emerged as a useful tool in medical diagnostics and health-related data interpretation, integrating diverse, heterogeneous data streams into a unified analytical framework. By leveraging complementary and redundant information across modalities, this approach mimics expert clinical reasoning and allows models to capture complex, non-linear biological interactions. This integration significantly enhances diagnostic accuracy, robustness, and generalizability compared to single-modality systems, ultimately bringing advanced AI closer to routine clinical practice (Stahlschmidt et al., 2022; Lipkova et al., 2022; Jandoubi and Akhloufi, 2025).

Combining multi-omics signatures, including genomics, epigenomics and transcriptomics, with various medical imaging modalities, such as computed tomography (CT), magnetic resonance imaging (MRI), and histopathology, has emerged as a highly promising frontier for the advancement of precision oncology (Marouf et al., 2025). The large volume of patient data and treatment options creates a complex decision process, driving the need for advanced analytical tools to support cancer care. Thus, AI methods have become universal in biomedical research, solving complex tasks like protein folding and radiology interpretation at the level of human experts (Unger and Kather, 2024).

In this review, relevant literature was identified through searches of PubMed, Scopus, and Google Scholar for English-language publications, with an emphasis on work from the past 5 years, using combinations of keywords including multimodal data integration, deep learning, machine learning, and precision oncology, together with cancer-type-specific terms. Representative studies were then selected to illustrate the principal data modalities, fusion strategies, and clinical applications discussed here, prioritizing recent, methodologically influential, and clinically relevant contributions over exhaustive coverage. We first examined each type of unimodal data of interest in precision oncology, spanning from the clinical data from electronic health records and the radiological and histopathological imaging data to the molecular profiles at the genomic, transcriptomic, epigenomic and proteomic level. Open-access data repositories which have been used for training ML models are also highlighted. In the second half of the article, we focused on the multimodal approach that is currently used in cancer management, exploring the developments made in the fields of ML and DL for multimodal data integration. Finally, we summarized in a comprehensive table the most cutting-edge AI applications successfully used for integrating multi-omics and multimodal data in precision oncology.

## 2 Types of data in precision oncology

Oncology data is broadly classified into three complementary categories: clinical, imaging, and molecular data (Tomczak et al., 2015). Although unimodal data sources, such as independent radiological images or clinical notes, have been the cornerstone of medical diagnostics, they ultimately fail to capture the complexity of human diseases. Therefore, managing complex conditions, such as cancer, requires a holistic phenotype and genotype analysis, which involves the integration of data from multiple modalities to ensure accurate diagnosis and treatment (Jandoubi and Akhloufi, 2025; Marouf et al., 2025).

Radiological imaging can be performed through several methods, such as CT, MRI and positron emission tomography (PET). Both radiology and histopathology provide the spatial and structural context, while multi-omics data, encompassing genomics, transcriptomics, proteomics, and/or epigenomics, offer profound molecular-level insights. Together, they facilitate a comprehensive evaluation of cancer phenotypes, correlating morphological features with biological mechanisms, with impactful implications in precision oncology since they provide personalized treatment opportunities (Acharya and Mukhopadhyay, 2024; Marouf et al., 2025).

To support this multimodal approach, a wide array of publicly available databases have been developed, ranging from comprehensive patient unimodal resources (e.g., comprising only imaging or genomics data) to resources that combine clinical records, medical images, and text annotations, all of which have been used for training ML models (Acharya and Mukhopadhyay, 2024; Haq et al., 2025). On the molecular and phenotypic level, foundational datasets encompassing genomics, transcriptomics, epigenomics, and proteomics, such as The Cancer Genome Atlas (TCGA) (Weinstein et al., 2013; The Cancer Genome Atlas Program (TCGA) - NCI, 2022), with matching radiological imaging (such as CT, MRI, and PET scans) provided by The Cancer Imaging Archive (TCIA) (Clark et al., 2013), are widely accessible. Beyond molecular characterization, modern multimodal datasets are increasingly bridging clinical phenotypes, imaging, and unstructured textual data. Key examples include PAD-UFES-20 (Pacheco et al., 2020), which pairs detailed clinical data with smartphone-captured skin lesion images, or the ROCO/ROCOv2 datasets, which couple radiology imaging with corresponding figure descriptions (Pelka et al., 2018; Rückert et al., 2024). The most comprehensive and consistently updated open-access databases used for training ML models are summarized alongside their respective modalities in Table 1.

**Table 1:** Detailed list of the datasets used to train machine learning models.

Dataset name	Reference (Author, Year/Official Source Link)	Data modalities	Primary application	Size
Thoracic imaging. Pulmonology				
MIMIC-CXR	(Johnson et al., 2019, 2024)	Chest X-rays, free-text reports	Automated chest radiograph interpretation, report generation	377,110 images, 227,835 studies, 65,379 patients
CheXpert	(Stanford AIMI Shared Datasets, n.d.)	Chest X-rays, text reports (with uncertainty labels)	Chest radiograph interpretation, automated pathology detection	224,316 images from 65,240 patients
RadFusion	(Zhou et al., 2021)	CT scans, structured EHR variables	Benchmark for multimodal pulmonary embolism detection	1,794 patients
PadChest	(Bustos et al., 2020; PADCHEST – BIMCV, n.d.)	Chest X-rays, multi-label annotated reports	Chest radiograph diagnosis	>160,000 images, >67,000 individuals
IU X-ray (Indiana Chest X-ray)	(Demner-Fushman et al., 2016)	Chest X-rays, radiology reports	Cross-modal retrieval, image captioning	8,121 scans, 3,996 reports
INSPECT	(Huang et al., 2023; (INSPECT, n.d.)	CT, EHR	Pulmonary embolism diagnosis and prognosis	19,438 patients
NIH Chest X-rays	(NIH Chest X-rays, n.d.)	Chest X-rays, 14 disease labels	Automated chest radiograph classification and deep learning benchmarking	112,120 X-ray images from 30,805 unique patients
Neuroimaging				
ADNI (Alzheimer’s Disease Neuroimaging Initiative)	(Zhang et al., 2022; Alzheimer’s Disease Neuroimaging Initiative, n.d.)	Structural MRI, PET, CSF biomarkers, cognitive assessments, genetics	Alzheimer’s disease (AD) progression, classification, dementia staging	>2,500 participants
NACC	(Gourdeau et al., 2026; Yang et al., 2026)	MRI, clinical, cognitive, genetic data	Dementia classification: AD, Frontotemporal Dementia (FTD), Lewy Body Dementia (DLB), Vascular Dementia (VaD)	>19,000 patients
BRATS	(Menze et al., 2015)	MRI (T1, T1Gd, T2, T2-FLAIR)	Brain tumor segmentation	8,160 MRI scans from 2,040 patients
OASIS-3	(LaMontagne et al., 2019; Open Access Series of Imaging Studies (OASIS), n.d.)	Brain MRI, PET scans, clinical and cognitive test data (text)	Dementia detection, Alzheimer’s progression modeling	1,098 subjects (longitudinal study)
Oncology				
TCGA (The Cancer Genome Atlas)	(Tomczak et al., 2015; The Cancer Genome Atlas Program (TCGA) - NCI, 2022)	Genomics, transcriptomics, epigenomics, histopathology, clinical data	Pan-cancer biomarker discovery, survival analysis, precision oncology, tumor molecular stratification	11,315 patients across 33 distinct tumor types (2.5 PB of data)
TCIA (The Cancer Imaging Archive)	(Clark et al., 2013; Welcome to The Cancer Imaging Archive, n.d.)	CT, MRI, PET, X-ray, histopathology, clinical data	Radiomics, radio-genomics, medical image segmentation, image-based deep learning, multimodal oncology	Massive repository (>50 million images across hundreds of collections), inherently linked to TCGA genomic cohorts
CCGA (The Circulating Cell-free Genome Atlas)	(Klein et al., 2021; GRAIL, Inc., 2023)	Blood cell-free DNA (cfDNA) methylation sequencing, clinical metadata	Multi-cancer early detection (MCED) via cfDNA	15,254 total participants
MSK-IMPACT	(Zehir et al., 2017; cBioPortal for Cancer Genomics, n.d.)	Targeted tumor NGS sequencing (>500 genes), clinical metadata	Precision oncology, survival analysis, therapy response prediction	>10,000 patients
CCLE (Cancer Cell Line Encyclopedia)	(Barretina et al., 2012; Cancer Cell Line Encyclopedia (CCLE), n.d.)	Cell line multi-omics (Expression, CNV, Mutations), drug screening profiles (text)	Drug sensitivity prediction, pre-clinical oncology modeling	947 cancer cell lines across 36 cancer types; pharmacological profiling for 24 drugs
SEER	(SEER incidence data - SEER Data & Software, n.d.)	Clinical data, demographic survival registry	Population-level outcomes, survival modeling, epidemiology	US cancer registry covering >47% of population
CPTAC	(Clinical Proteomic Tumor Analysis Consortium (CPTAC), n.d.)	Proteomics, genomics, transcriptomics, imaging, clinical data	Proteogenomics, linking omics with imaging/clinical outcomes	Multiple specific cancers (breast, colon, ovarian, lung, etc.)
Histopathology				
MoNuSeg	(Kumar et al., 2020; MoNuSeg - Grand Challenge, n.d.)	H&E stained histopathology images, pixel-level nuclear masks	Multi-organ nuclei segmentation, digital pathology analysis	30 images containing >21,000 annotated nuclei across 7 organs
PathVQA	(Naseem et al., 2023)	Histopathology images, structured Question-Answering (QA) text pairs	Medical Visual Question Answering (VQA), oncology diagnostic support	4,998 pathology images coupled with 32,799 unique Question-Answer pairs
CAMELYON	(Litjens et al., 2018)	Lymph node H&E Whole Slide Images (WSI)	Breast cancer metastasis detection, patch-level classification	399 WSIs (Cam16)/1,000 WSIs (Cam17)
Dermatology				
HAM10000	(Tschandl et al., 2018)	Dermatoscopic images, clinical metadata (text)	Skin lesion classification	10,015 images
PAD-UFES-20	(Pacheco et al., 2020)	Clinical images, patient metadata (age, sex, skin type, lesion location)	Skin lesion diagnosis (e.g., melanoma, carcinoma)	2,298 images, 1,373 patients
Ophthalmology				
FFA-IR	(Li M. et al., 2021)	Fundus images, bilingual reports, lesion annotations	Retinal disease diagnosis, descriptive medical report generation	1,330 samples
General intensive care. Population Data				
MIMIC-III/MIMIC-IV	(Johnson et al., 2016, 2023)	Electronic Health Records (structured, time-series), clinical notes	ICU risk prediction (mortality, sepsis), disease diagnosis generation	>53,000 to >383,000 admissions
UK Biobank (UKB)	(Sudlow et al., 2015)	MRI, CT, ultrasound, bone density scan (DXA), genomics, clinical/lifestyle EHR	Multimodal disease risk prediction, population health tracking	500,000 + participants
Multimodal medical VQA and benchmarks				
MedICaT	(Allen Institute for Artificial Intelligence, 2020; Subramanian et al., 2020)	Medical figures, captions, inline references	Scientific radiology figure interpretation, subfigure alignment	217,060 figures from 131,410 papers
ROCO/ROCOv2	(Pelka et al., 2018; Rückert et al., 2024)	Radiology images (X-ray, CT, MRI, ultrasound), text (captions, UMLS medical concepts/CUIs)	Cross-modal retrieval, medical image captioning (report generation), vision-language grounding, multi-label image classification	ROCO (2018): 81,825 radiology images
				ROCOv2 (2024): 79,789 highly-filtered radiology images
SLAKE/SLAKE-VQA	(Liu et al., 2021)	X-ray, CT, MRI, QA text, medical knowledge graphs	Medical Visual Question Answering (Med-VQA)	642 images, 14,028 QA pairs
PMC-VQA	(Zhang et al., 2024)	Radiology/pathology/biomedical scans, QA text	Medical Visual Question Answering	227,000 VQA pairs across 149,000 images
MedTrinity-25 M	(Xie et al., 2025)	CT, MRI, X-ray, Ultrasound, Dermoscopy (10 modalities), text	Medicine foundation models, multigranular annotations	25 million images
EHRXQA	(Bae et al., 2023)	Chest X-rays, EHR, QA pairs	QA-focused dataset for multimodal reasoning	457,400 + QA pairs

### 2.1 Clinical data

Conventionally, medical diagnosis has relied on patient history and physical examinations. However, this process tends to be challenging due to complex disease mechanisms and nonspecific symptoms. In order to increase the efficiency of the diagnostic process, medical history, physical examinations, and laboratory results are saved in centralized EHRs in most countries. These digital databases provide a comprehensive view of the clinical and paraclinical data, encompassing past diagnoses and treatments, to enable clinicians to thoroughly understand the underlying disease (Wekesa and Kimwele, 2023; Waqas et al., 2024). Within the EHR, longitudinally-followed data, such as repeated lab values or physical attributes recorded over time, are crucial for monitoring disease progression and evaluating continuous changes within the evolution of the patients (Waqas et al., 2024).

With the aid of AI tools, such as semi-automatic and automatic natural language processing (NLP) frameworks, diagnostic efficiency can be enhanced. For example, such a semi-automatic NLP model has been developed for mapping unstructured patient narratives to a causal chain of abnormal states, providing a foundation for the deep phenotyping necessary in precision medicine (Ma et al., 2017). Concurrently with text-based processing, AI has also revolutionized the objectification of physical attributes during clinical examinations. For instance, Face2Gene is a deep learning framework that analyzes facial photographs to detect dysmorphic features and distinctive facial phenotypes associated with rare genetic disorders, thereby accelerating syndromic classification that is sometimes challenging for human clinicians at first glance (Gurovich et al., 2019).

Similar to how human physicians rely on diverse types of information, automated diagnostic systems that successfully integrate clinical EHR data, such as patient demographics and previous diagnoses, with paraclinical data (including imaging and molecular biomarkers) can achieve superior performance compared to systems relying on isolated data streams, yielding highly clinically relevant models (Huang et al., 2020).

### 2.2 Imaging data. Radiology and histopathology findings

Medical imaging techniques are essential in modern clinical practice and comprise a significant proportion of medical data, providing complementary information for disease diagnosis and treatment evaluation (Haq et al., 2025; Marouf et al., 2025). These modalities are crucial in oncology and are broadly divided into macroscopic (radiological imaging) and microscopic (digitized histopathology) (Rowe and Pomper, 2022; Waqas et al., 2024).

Radiological techniques, such as ultrasound, X-rays, CT, MRI, and PET, provide the anatomical and functional views needed to detect abnormal structures, determine tumor extent, and assess treatment efficacy (Rowe and Pomper, 2022; Wekesa and Kimwele, 2023; Waqas et al., 2024; Haq et al., 2025). Specifically, PET captures functional metabolic activity, while CT and MRI offer detailed anatomical and soft tissue visualization (Marouf et al., 2025). Owing to the fact that the manual interpretation of complex scans is inherently laborious and vulnerable to human error, DL models are increasingly utilized for providing fast and highly precise diagnostic predictions (Wekesa and Kimwele, 2023). For example, in the context of screening and diagnostic triage, DL models have been used in cardiology for automatic detection of coronary artery stenosis and calcified plaques on Coronary Computed Tomography Angiography (CCTA) imaging, achieving up to 96% accuracy and performing on par with trained physicians (Edpuganti et al., 2025), while in the field of oncology, Heenaye-Mamode Khan et al. (2021) developed a deep convolutional neural network (CNN) framework that performs automated multi-class classification of breast cancer abnormalities using raw mammography scans alone, underlining the utility of DL in distinguishing subtle structural anomalies. In a similar manner, for downstream patient management, Hu et al. (2022) have developed a deep learning framework that is able to offer predictions on glioblastoma survival by automatically extracting high-dimensional features from multi-parametric MRI scans, a process that overcomes the need for laborious manual feature extraction, with an estimated accuracy of 0.745. Another important clinical milestone was the release of Sybil (Mikhael et al., 2023) in 2023, which is a DL model developed to predict future lung cancer risk up to 6 years in advance directly from a single low-dose chest CT scan, identifying subtle micro-structural patterns invisible to the human eye without requiring manual radiologist annotations.

Beyond macroscopic radiology, histopathology investigates the precise morphology of a tumor and serves as a fundamental pillar of precision oncology, given that all solid tumors require cytological diagnosis for guiding clinical treatment decisions (Unger and Kather, 2024). Through the development of modern digital pathology scanners, traditional tissue samples obtained via biopsy are now transformed into whole slide images (WSI) (Unger and Kather, 2024; Al-Zoghby et al., 2025). These digitized images reveal detailed structural changes in cancer cells, enabling clinicians to accurately diagnose and subtype cancer with unprecedented clarity (Waqas et al., 2024). DL models are able to extract subtle patterns from WSIs in order to perform various tasks, such as predicting prognosis, facilitating personalized therapy decisions based on mutational status, drug response, and overall survival predictions. Such an example is the MC-TMB framework proposed by Chen et al. (2023), which analyzes hematoxylin and eosin (H&E)-stained WSIs in approximately 4 min. The MC-TMB framework can predict the tumor mutational burden (TMB) based solely on histopathological findings, offering a time-efficient and accessible alternative to traditional genomic sequencing for identifying potential immunotherapy candidates.

Ultimately, to seamlessly deploy these heavy radiological and histopathological imaging frameworks into daily clinical practice, modern oncology workflows rely on highly efficient neural network architectures, such as ShuffleNet (Zhang et al., 2017), which aims to reduce computational complexity for fast and accurate diagnostic processing on standard hospital computers.

### 2.3 Molecular and multi-omics signatures. Biomarkers

Omics technologies are high-throughput techniques that investigate biological molecules, providing crucial information about the underlying genetic alterations that occur in cancer cells. These disciplines include genomics for decoding DNA sequences, transcriptomics for analyzing RNA expression, proteomics for studying peptides and proteins, and metabolomics for identifying small-molecule compounds. Together, multi-omics datasets can provide comprehensive perspectives for addressing complex biomedical problems, including disease diagnosis, prognosis, and therapeutic interventions (Wekesa and Kimwele, 2023; Waqas et al., 2024; Al-Zoghby et al., 2025).

Molecular signatures identified through various wet-lab molecular techniques act as biomarkers that are broadly classified as diagnostic, predictive, or prognostic. Diagnostic biomarkers, such as prostate-specific antigen (PSA) values, serve as the first line of cancer detection. Evolving from early ML applications for automated white blood cell classification via accessible no-code platforms, such as Teachable Machine released in 2017 (Hoseini and Dewar, 2024), the push for early, non-invasive diagnosis has shifted toward targeting circulating tumor components in the blood through the novel concept of liquid biopsies. A milestone was the development of CancerSEEK (Cohen et al., 2018) in 2018, a multi-analyte test that combines the mutation tracking of cell-free DNA (cfDNA) with protein biomarker levels to detect and locate eight common cancer types before symptoms appear. A year later, it was followed by the DELFI (Cristiano et al., 2019) framework, which uses ML for genome-wide cfDNA fragmentation pattern analysis, taking advantage of the fact that cancer cells shed DNA into the bloodstream in uniquely abnormal configurations. Furthermore, the combined use of DL and methylation information has proven highly effective for non-invasive cancer detection, through models such as cfMethylPre, which is able to analyze circulating cfDNA methylation profiles, while tools like MSI-XGNN leverage microsatellite instability using combined methylation and transcription biomarkers (Pălăștea and Bucur, 2025). Predictive biomarkers can determine cancer aggressiveness or anticipate treatment responses, such as KRAS mutations which can indicate resistance to specific therapies. Finally, prognostic biomarkers focus on estimating the risks associated with clinical outcomes, including overall survival and disease recurrence (Lipkova et al., 2022).

Due to the uniquely encoded molecular characteristics of tumors, clinical genomics is vital for delivering precision oncology. While histopathology addresses phenotypic changes, genomics explores the specific disease genotype, offering insights regarding genomic instability and mutation status (Unger and Kather, 2024). In the field of pharmacogenomics, drug response predictions often rely on cancer cell line cultures, enabling genome-wide association studies to simultaneously screen a broad number of cancer-drug pairs (Unger and Kather, 2024). Additionally, identifying the abnormal expression of non-coding RNAs (ncRNAs) provides vital biological candidates for understanding disease mechanisms and advancing targeted drug discovery (Wekesa and Kimwele, 2023). Because genomic information is typically extracted after an initial histological diagnosis, DL applications in clinical genomics focus primarily on advanced tasks, such as novel biomarker discovery and drug-response prediction, rather than streamlining basic diagnostic workflows (Unger and Kather, 2024). Extracting meaningful feature information from complex, unlabeled multi-omics datasets remains a challenging representation learning problem, but advanced computational tools are continually being developed to enhance feature learning and predictive abilities across these varied biological views (Wekesa and Kimwele, 2023). An exponent of this computational progress is DeepVariant (Poplin et al., 2018), a specialized unimodal framework that successfully automates basic data preparation by treating DNA sequencing data as an image, operating with deep CNN for genetic variant detection.

## 3 AI and machine learning for multimodal data integration

**Figure 1:**
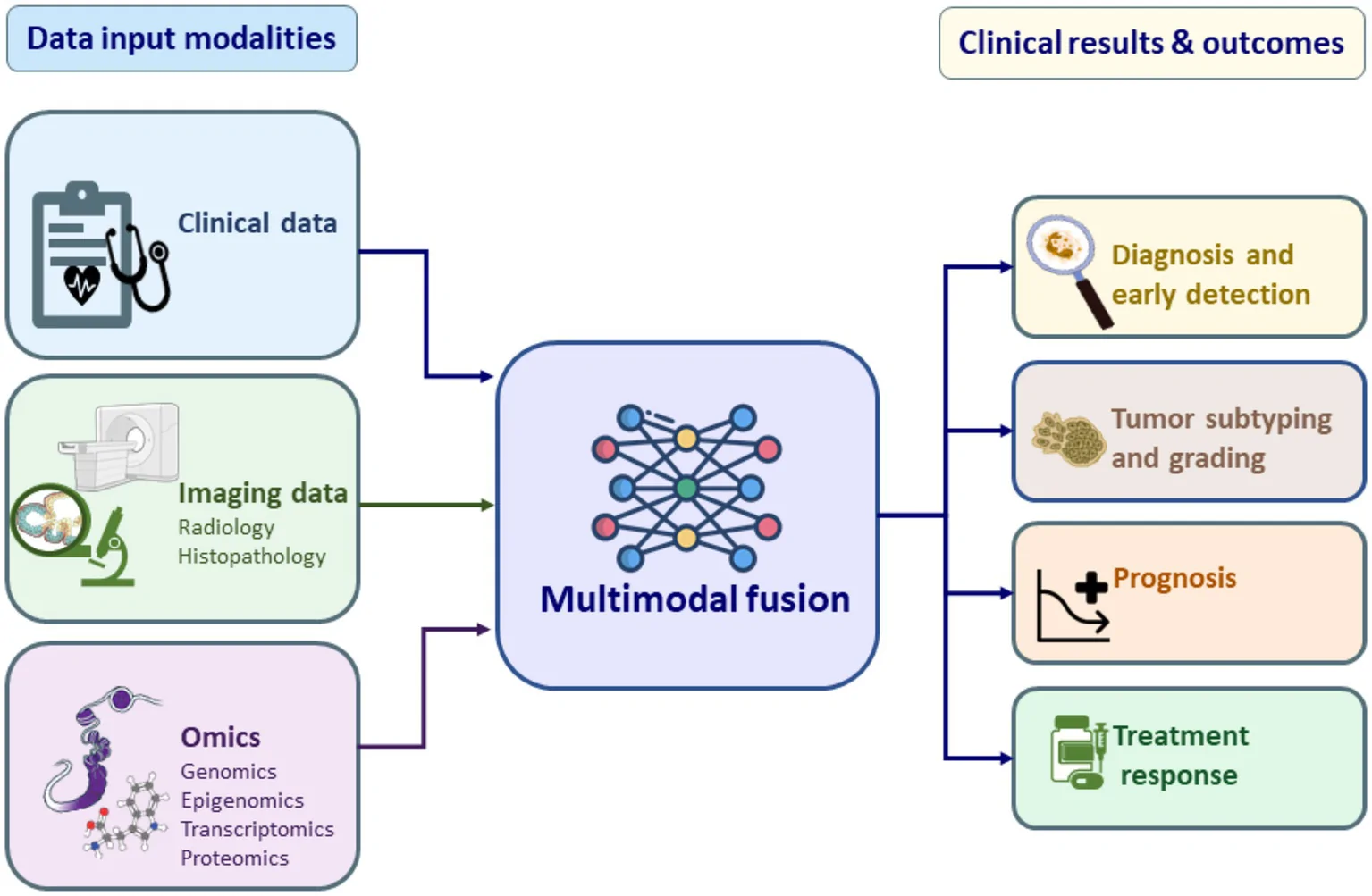
Overview of integrated multimodal AI (MMAI) for precision oncology. This diagram illustrates how diverse clinical, multi-scale imaging, and molecular omics data are processed through deep learning and machine learning strategies to automate diagnostic workflows, forecast prognosis, predict treatment outcomes, and discover novel biomarkers. This figure was created in PowerPoint based on concepts and references from multiple published studies, including Haq et al. (2025) and Dellamonica et al. (2025).

DL approaches currently play an important role in oncology diagnosis, prognosis, and treatment, reducing wet-lab procedural time and costs (Wekesa and Kimwele, 2023). The development shifted from traditional ML pipelines, which often use unimodal data streams that tend to fragment the complexity of the cases, increasing the risk of delayed interventions and errors, toward multimodal AI (Al-Zoghby et al., 2025; Jandoubi and Akhloufi, 2025). MMAI fuses diverse and distinct data modalities, such as radiology, pathology, genomics, and electronic health records, constructing a holistic view that mimics the comprehensive decision-making process of expert clinicians (Haq et al., 2025) (Figure 1). Ultimately, this type of integration aims to bridge fragmented data in order to increase diagnostic accuracy, reliable prognosis, and the discovery of novel biomarkers, outcomes which would be significantly more difficult or even impossible to achieve using a single modality (Lipkova et al., 2022).

There are many specific DL models employed within these complex pipelines. For grid-topology data-like images, CNNs excel at capturing spatial hierarchies, while sequential data can be processed by Recurrent Neural Networks (RNNs). Fully Connected Neural Networks (FCNNs) learn non-spatial hierarchies that are suitable for tabular data, and Autoencoders (AEs) are utilized for dimensionality reduction. Operating on graph structures, Graph Neural Networks (GNNs) leverage relationships and interdependencies, whereas Transformers (TF) and Vision Transformers (ViTs) utilize self-attention mechanisms, excelling in parallel processing and handling sequential or visual data. A general oncology pipeline typically comprises feature extraction, fusion, and prediction, where DL models encode imaging and multi-omics features (Guarrasi et al., 2025; Marouf et al., 2025).

Building on these self-attention mechanisms, Transformer-based models have become particularly influential for multimodal integration, as they are able to model long-range dependencies across heterogeneous inputs. Beyond standard self-attention, several models leverage cross-modal and co-attention designs, in which one modality guides the interpretation of another. For instance, MCAT (Chen R. J. et al., 2021) utilizes genomic-guided co-attention, whereas CATfusion (Hu et al., 2025) employs a cross-attention fusion network to integrate imaging and multi-omics data. Hierarchical, multi-scale variants have also emerged, such as SMuRF (Song et al., 2025), which utilizes a Swin-Transformer backbone to aggregate features across spatial resolutions in gigapixel whole-slide images. Furthermore, graph-enhanced approaches, such as Pathomic Fusion (Chen et al., 2022a), combine attention with relational structures to link morphological and molecular features.

A distinct and rapidly expanding category is represented by foundation models, which are pretrained on large, often unlabeled datasets to learn transferable representations that can be adapted to numerous downstream tasks with minimal task-specific retraining. In oncology, these include whole-slide pathology foundation models, such as TITAN (Ding et al., 2025), as well as foundation model-driven embedding frameworks like HONeYBEE (Tripathi et al., 2025), which integrates clinical, imaging, and genomic data at scale. Closely related are Vision-Language Models (VLMs), which align images with free text to enable tasks such as zero-shot classification, automated report generation, and visual question answering. Representative examples in oncology include CONCH (Lu et al., 2024), a contrastive vision-language model for computational pathology, and a Vision-Language Transformer designed for interpretable pathology visual question answering (Naseem et al., 2023). The comparative strengths and limitations of these architecture families, including their data and computational demands, scalability, and interpretability, are examined in Section 5.1.

Large language models (LLMs) represent a further, text-centric branch of this generative paradigm, and are increasingly explored in oncology for tasks such as clinical decision support, literature synthesis, biomedical knowledge extraction, the summarization of pathology and radiology reports, and clinical trial matching, as well as for integration within multimodal systems. Their clinical adoption nonetheless remains constrained by well-documented limitations, including hallucination, limited factual reliability, and the need for domain adaptation, in addition to the privacy and regulatory considerations examined in Section 5.2 (Al-Zoghby et al., 2025).

Core to multimodal learning are data fusion strategies, which determine how and when different data sources are integrated into a model (Jandoubi and Akhloufi, 2025). This integration leverages complementary information to uncover deep, non-linear biological interactions and bridges the genotype–phenotype gap to improve prognostic accuracies (Wekesa and Kimwele, 2023; Guarrasi et al., 2025).

Early fusion strategies combine raw features or extracted features from different modalities into a single feature vector before input to the final model (Huang et al., 2020; Jandoubi and Akhloufi, 2025). This approach exploits the joint distribution of heterogeneous features, also increasing dimensionality, and requires careful data processing and normalization (Marouf et al., 2025). Applications include the integration of similar imaging modalities, such as multi-view ultrasound or structural and functional scans, and integrating imaging data with electronic medical records (EMRs) (Lipkova et al., 2022).

Late fusion strategies process each modality independently and combine individual predictions or embeddings at the decision level. This flexible strategy allows modality-specific specialization for heterogeneous data but may fail to capture subtle cross-modal interactions that emerge only at the feature level (Al-Zoghby et al., 2025; Marouf et al., 2025). Examples involve combining imaging scans with non-imaging inputs, such as blood tests, and integrating genomics and histology profiles for survival prediction (Lipkova et al., 2022).

Intermediate (feature-level or joint) fusion processes each data modality with its own specialized neural network branch to extract latent feature representations, merging them within a shared common layer (Huang et al., 2020; Al-Zoghby et al., 2025). By striking a balance between early and late strategies, this approach manages dimensionality and heterogeneity while also enabling the model to learn complex, non-linear interactions before generating a final prediction (Guarrasi et al., 2025). It is widely considered an effective strategy, and it has been extensively used in diverse imaging and multi-omics integration tasks (Lipkova et al., 2022).

Hybrid fusion is an advanced strategy that combines multiple techniques, such as early, intermediate, and late fusion, within a single unified architecture that leverages the distinct strengths of each1. It simultaneously uses feature interactions and decision independence to enhance predictive robustness and accuracy, particularly when handling highly heterogeneous medical data, such as electronic health records or imaging (Al-Zoghby et al., 2025). Unlike single-strategy methods, hybrid models often establish hierarchical relationships between modalities in order to capture both low-level correlations and high-level abstractions (Jiao et al., 2024).

## 4 Clinical applications in AI-driven oncology

The integration of AI into healthcare has advanced rapidly in recent years, with multimodal approaches emerging as promising tools for improving diagnostic accuracy and clinical decision-making (Jandoubi and Akhloufi, 2025). By synergizing clinical, imaging, and molecular data, these technologies significantly accelerate the process of disease detection (Wekesa and Kimwele, 2023), while also providing a novel way of extracting insights from raw data, which can augment and potentially replace certain aspects of traditional evaluation workflows. In precision oncology in particular, the clinical applications of these multimodal models can be broadly categorized into 3 groups of applications according to the clinical utility, namely tools for diagnosis and early detection, tumor subtyping and grading, or prognosis and treatment response (Unger and Kather, 2024). In Table 2, we have summarized a series of AI tools currently utilized for multimodal data integration in the field of oncology. We classified these applications into two main categories: foundational, large-scale frameworks designed for broad multimodal processing, and custom pipelines, representing smaller architectures explicitly engineered to solve specific tasks.

**Table 2:** Overview of AI applications for multimodal data integration in precision oncology.

Tool	Cancer type	Data modalities	Architecture	Fusion strategy	Explainability*	Clinical applications	Reported performance	Reference
Open-source foundational frameworks								
iClusterPlus	Pan-cancer	Genomics, Transcriptomics, Epigenomics, Proteomics	R/Bioconductor ML framework	Intermediate (joint latent variable mode)	Feature selection (Lasso); no statistical inference for selected features	Diagnosis, subtyping, prognosis estimation	Not specifically reported; demonstrated successful identification of clinically relevant subtypes in TCGA lung, endometrial, and gastric cohorts	(Mo et al., 2013; Mo and Shen, 2026)
MOFA+	Pan-cancer	Epigenomics, Transcriptomics and Clinical data	Bayesian factor analysis	Intermediate (latent factors)	Feature loadings (sparsity-enforced), allowing biological pathway enrichment	Patient stratification, subtyping, prognosis estimation, survival prediction	BRCA subtyping (F1: 0.752; CHI: 42.42); outperformed deep learning baselines (MoGCN) in feature selection and cluster definition	(Argelaguet et al., 2020; Omran et al., 2025)
MOGONET	Pan-cancer (validated on glioma, breast and kidney cancer cohorts)	Genomics, Transcriptomics, Epigenomics, Proteomics, Metabolomics, and Clinical data	GCN (view-specific + consensus graph)	Late (VCDN/ consensus graph)	Feature importance ranking; identifies biomarkers through graph node and edge attribution analysis	Diagnosis (biomarker identification); subtyping, prognosis estimation, survival risk stratification	Demonstrated superior classification over conventional unimodal and late-fusion baselines. Benchmarks: BRCA subtyping (ACC 0.829, F1 0.825); LGG grading (ACC 0.816, AUC 0.840); ROSMAP (ACC 0.815, AUC 0.874).	(Wang et al., 2021)
Seurat v4	Pan-cancer	Single-cell Transcriptomics, Single-cell Epigenomics, and Single-cell Proteomics	WNN graph	Intermediate (graph)	Modality weights (cell-specific)	Subtyping (TME characterization); prognosis and treatment response (immunotherapy-resistance biomarkers)	Not reported	(Hao et al., 2021)
MONAI	Pan-cancer	Radiology, Clinical Data and Functional data (EEG)	PyTorch-based ecosystem	Framework-dependent; (Supports modular implementation of Early, Intermediate and Late fusion)	Grad-CAM, Grad-CAM++, Occlusion sensitivity, and Smoothgrad	Early detection, subtyping, AI-driven risk assessment	Foundation for models achieving 99.2% DSC in breast delineation and 27% FPR reduction (0.41 to 0.30) in lung cancer screening compared to Lung-RADS	(Cardoso et al., 2022; MONAI, n.d.)
MUON	Pan-cancer	Transcriptomics, Epigenomics, Proteomics and Clinical data	Modular Python framework	Intermediate (joint)	Cross-layer feature importance ranking	Tumor subtyping and grading (TME mapping), survival risk estimation	Not reported	(Bredikhin et al., 2022)
Porpoise	Pan-cancer	Histopathology (WSI), Genomics, Transcriptomics	Attention-based MIL for images and SNN for molecular features	Intermediate (attention)	Attention heatmaps for tissue regions; IG with SHAP-styled attribution decision plots for molecular feature importance	Biomarker discovery, subtyping, survival outcome prediction	Overall mean C-index 0.644 across 14 TCGA cohorts; demonstrated significant performance in KIRP (C-index 0.816) and LGG (C-index 0.808)	(Chen et al., 2022b)
CustOmics	Pan-cancer	Genomics, Transcriptomics, Epigenomics	Customizable AE	Intermediate (latent)	Feature importance ranking	Diagnosis, subtyping, survival modeling	Not reported	(Benkirane et al., 2023)
PathOmics	Pan-cancer	Histopathology (WSI), Genomics, Transcriptomics	Multimodal transformer (unsupervised pretraining)	Intermediate (attention)	Attention maps	Diagnosis, survival prediction	Not reported	(Ding et al., 2023)
CONCH	Pan-cancer	Histopathology (WSI), Clinical text	Contrastive vision-language foundation model	Intermediate (contrastive alignment)	Attention weights	Diagnosis, subtyping, prognosis	Zero-shot subtyping accuracy: 90.0% (NSCLC), 89.3% (RCC); superior cross-domain generalization compared to traditional supervised baselines	(Lu et al., 2024)
HONeYBEE	Pan-cancer	Clinical data, Radiology, Histopathology (WSI), Genomics, Proteomics	Foundation-model-driven embedding framework	Hybrid (adaptive); incorporates cross-modal attention and adaptive fusion mechanisms	Cross-modal attention weights	Diagnostic fusion; cross-modal subtyping; prognosis estimation	Not specifically reported; shows improved prognostic accuracy over traditional unimodal models	(Tripathi et al., 2025)
Custom pipelines								
Pathomic Fusion	Pan-cancer (validated on Glioma and CCRCC cohorts)	Histopathology (WSI), Genomics, Transcriptomics	CNN (patch-level), GCN (cell-graph level), and SNN (genomic-level)	Intermediate (attention/tensor): uses Kronecker product to model pairwise feature interactions with a gating-based attention mechanism	Attention (gating)	Subtyping; survival prediction	C-index 0.826 (Glioma) and 0.720 (CCRCC); benchmarks in LGG (0.817), BRCA (0.717), and LUSC (0.618) cohorts	(Chen et al., 2022a)
MCAT	Pan-cancer	Histopathology (WSI), Genomics	Transformer, genomic-guided co-attention	Intermediate (co-attention)	Attention (co-attention)	Survival prediction	Mean C-index 0.683–0.695 (pan-cancer/joint); benchmarks in GBMLGG (0.835), BLCA (0.672), and LUAD (0.659)	(Chen R. J. et al., 2021)
KDBTC	Breast cancer	Clinical data, Imaging reports (text)	HA-BiRNN and tree-structured GRU	Intermediate (text encoding)	Hierarchical attention weights	Breast tumor subtyping	Not specifically reported; outperformed conventional RNN-based and single-report baselines in breast tumor classification	(Chen D. et al., 2021)
MoGCN	Breast (BRCA) and kidney (KIPAN) cancer	Genomics, Transcriptomics, Proteomics	GCN pipeline integrated wiith an autoencoder and SNF	Intermediate (graph + latent)	Sensitivity analysis (feature importance ranking based on standard deviation and connection weights) and (PSN) visualization	Biomarker discovery, breast tumor subtyping	BRCA subtyping: Accuracy 0.898, F1 0.902; KIPAN subtyping: Accuracy 0.977, F1 0.977; demonstrated superior performance over SVM, RF, and standard DNN baselines	(Li et al., 2022)
GAE-LGA	Pan-cancer	Transcriptomics and molecular profile features	Graph AE	Intermediate (graph)	Adjacency matrix reconstruction	Biomarker discovery, Prognosis (identifying functional molecular correlates and lncRNA-PCG associations)	Not reported	(Gao et al., 2022)
DyAM	NSCLC	Radiology (CT imaging), Histopathology (PD-L1 IHC), Genomics and Clinical data	Dynamic attention-based MIL	Intermediate (attention)	Partial risk scores and attention weights/shares	Prognosis and treatment response (predicting response to PD-(L)1 blockade immunotherapy)	AUC 0.80 (immunotherapy response prediction); C-index 0.74 (multivariable model); significantly outperformed unimodal TMB (AUC 0.61) and PD-L1 IHC (AUC 0.73)	(Vanguri et al., 2022)
RadGenNets	NSCLC	Radiology (PET scans), Genomics, Clinical data	CNNs for imaging and DNNs for structured molecular and clinical data	Intermediate (joint)	Not specified	Diagnosis and early detection (gene mutation prediction); prognosis	Not reported	(Tripathi et al., 2022)
Multimodal DL (NSCLC)	NSCLC	Radiology (3D whole-body FDG PET) and Clinical data	ResNet3D-34 (imaging branch) and DeepSurv Multilayer Perceptron (clinical branch)	Intermediate (joint)	Output-level interpretability (Individualized survival curve visualization and median residual life expectancy estimation)	NSCLC prognosis and overall survival prediction	C-index 0.756, MAE 399 days; significantly outperformed unimodal clinical CPH (C-index 0.747, MAE 583 days) and PET-only ResNet3D (C-index 0.749, MAE 405 days) models	(Oh et al., 2023)
DeepFusionCDR	Pan-cancer	Transcriptomics, Genomics, Epigenomics and Drug Chemical Structures	Molecule-specific transformers (for drug structures) paired with unsupervised contrastive learning for multi-omics integration	Intermediate (contrastive)	Self-attention weights	Treatment response (cancer drug-response prediction)	Not specifically reported; demonstrated enhanced predictive performance over standard unimodal and early-fusion drug-response baselines	(Hu et al., 2024)
SMuRF	HPV-associated oropharyngeal SCC	Radiology (CT imaging), Histopathology (multi-region WSIs)	Swin-transformer	Intermediate (attention)	Integrated Gradients for quantifying radiology importance; Attention heatmaps for localizing prognostic morphological features in WSIs; SHAP values for global variable importance	Grade classification, outcome prediction	C-index 0.79–0.81 (DFS prediction); AUC 0.74–0.75 (Tumor grade classification); demonstrated HR 17 (*p* < 0.0001) for DFS in multivariable analysis	(Song et al., 2025)
CATfusion	Pan-cancer	Histopathology (WSI), Genomics, Epigenomics, Transcriptomics	Transformer architecture utilizing TabAE for self-supervised genomic feature extraction and CTransPath for image mosaic	Intermediate (cross-attention)	Attention-based visualization	Subtyping, survival prediction, treatment response	Overall mean C-index 0.668 and survival AUC 0.628 (31 cancer types); highest benchmarks in TCGA-KIRC (C-index 0.87), LGG (0.849), and ACC (0.83)	(Hu et al., 2025)
UMPSNet	Pan-cancer	Clinical data, Histopathology (WSI), Genomics	OT-based attention and GMoE	Intermediate (attention)	Attention (OT-attention weights and Grad-CAM)	Pan-cancer survival outcome prediction	Mean C-index 0.725 (TCGA internal validation); Zero-shot C-index 0.652 (external validation on pancreatic cancer without fine-tuning)	(Zhang et al., 2025)
TITAN	Pan-cancer	Histopathology (WSI), Pathology reports (text)	Multimodal whole-slide foundation model using a ViT backbone; employs iBOT for visual pretraining and CoCa for vision-language alignment	Intermediate (cross-modal alignment)	Attention heatmaps	Diagnosis and early detection (rare disease/cancer retrieval), subtyping, survival outcome prediction	Balanced accuracy 0.781 (morphological classification); C-index 0.716 (survival prediction); outperformed PRISM in rare cancer retrieval by 14.8%	(Ding et al., 2025)
Radiogenomics ML (glioblastoma)	Glioblastoma	Imaging (MRI-based radiomic features), Genomics	Classical ML (RF, SVM, XGBoost, LR, Extra Trees)	Early (feature-level)	Feature-based (tree importance)	Diagnosis (predicting oncogenic signaling pathway activity) and subtyping	Not reported	(Ahanger et al., 2025)
Histopathology + multi-omics ML pipeline	Glioblastoma	Histopathology, Genomics, Transcriptomics, Proteomics	Classical ML (RF + WGCNA)	Early (feature-level)	Feature importance and module analysis	Diagnosis (molecular feature prediction), survival prediction	Not reported	(Huang et al., 2025)
PS-CRC	Colorectal cancer	Histopathology (WSI), Transcriptomics	DL based MIL	Intermediate (attention-MIL)	SHAP, Grad-CAM	Overall/disease-free survival; adjuvant chemotherapy benefit	Not reported	(Lou et al., 2025)
ViT + immune-gene gastric pipeline	Gastric cancer (specifically MSS)	Histopathology (WSI), Transcriptomics	ML gene signature combined with ViT models	Hybrid	Attention mechanisms	Subtyping, predicting survival and immunotherapy response	Not reported	(Ye et al., 2025)
GNPC	Nasopharyngeal carcinoma	Histopathology (WSI), Clinical data	GNN framework	Intermediate (graph)	Not specified	Prognosis estimation (risk stratification for distant metastasis, overall survival, and local recurrence)	Not reported	(Zhou et al., 2025)
PaGeFin	Pan-cancer (experimentally validated on oral cavity squamous cell carcinoma)	Histopathology, Epigenomics	Cross-modal correlative pipeline; utilizes SpaTIL to extract morphological features and MACS2, BEDTools, and MultiCOV for ATAC-seq peak analysis	Intermediate (joint)	Cross-modal association mapping	Diagnosis, subtyping, risk stratification for disease-free survival and identification of therapeutic targets	Not reported	(Monabbati et al., 2025)
DeepTFtyper	SCLC	Histopathology (WSI), Proteomics	Interpretable morphology-aware GNN	Intermediate (graph)	Interpretable by design (utilizes a morphology-aware graph structure to ensure biological transparency in translating images to molecular subtypes)	SCLC molecular subtyping, prognosis estimation	AUC 0.70–0.83 (validated for 4 molecular subtypes via cross-validation)	(Li X. et al., 2025)
Counterfactual co-attention transformer	Pan-cancer	Histopathology (WSI), Genomics	Bidirectional co-attention transformer	Intermediate (co-attention)	Counterfactual explanations	Histology-genomic cancer risk stratification	Not reported	(Ji et al., 2025)
Transcriptomics profiling (soft tissue)	Soft tissue tumors (sarcomas)	Clinical data, Genomics, Transcriptomics	Classical ML + unsupervised clustering	Early (feature-level)	Morphologic-molecular mapping	Tumor classification; prognosis estimation	Not reported	(Hofvander et al., 2026)
HSFSurv	Pan-cancer	Histopathology (WSI), Genomics	DL hybrid-supervision	Hybrid	Not specified	Survival prediction	Not reported	(Fu et al., 2026)

* Explainability: Refers to the analytical frameworks and visualization techniques used to render the internal logic of high-dimensional “black box” models transparent to human clinicians. AE, autoencoder; ACC, adrenocortical carcinoma; AI, artificial intelligence; ATAC-seq, assay for transposase-accessible chromatin sequencing; AUC, area-under-curve; BLCA, bladder urothelial carcinoma; BRCA, breast invasive carcinoma; CNN, convolutional neural network; CT, computed tomography; DFS, disease-free survival; DL, deep learning; FDG PET, Fluorodeoxyglucose Positron Emission Tomography; GAE, graph autoencoder; GBMLGG, glioblastoma multiforme and low-grade glioma; GCN, graph convolutional network; GMoE, guided mixture of experts; GNN, graph neural network; Grad-CAM, gradient-weighted class activation mapping; GRU, gated recurrent unit; HA-BiRNN, hierarchical attention bidirectional recurrent neural network; KIRC, kidney renal clear cell carcinoma; KIRP, kidney renal papillary cell carcinoma; LGG, lower grade glioma; lncRNA, long non-coding RNA; LR, logistic regression; LUAD, lung adenocarcinoma; MIL, multiple-instance learning; ML, machine learning; MRI, magnetic resonance imaging; MSS, microsatellite stable; NSCLC, non-small-cell lung cancer; OT, optimal transport; PCG, protein-coding gene; PD-L1 IHC, Programmed Death-Ligand 1 Immunohistochemistry; PET, positron emission tomography; RCC, renal cell carcinoma; RF, random forest; SCLC, small cell lung cancer; SCC, squamous cell carcinoma; SHAP, Shapley additive explanations; SNF, similarity network fusion; SpaTIL, spatial tumor-infiltrating lymphocyte architecture; SVM, support vector machine; TCGA, The Cancer Genome Atlas; TME, tumor microenvironment; ViT, vision transformer; WGCNA, weighted gene co-expression network analysis; WNN, weighted nearest neighbor; WSI, whole-slide imaging; XGBoost, extreme gradient boosting.

### 4.1 Diagnosis and early detection

Prior to any case-management decisions, it is imperative to provide an accurate diagnosis. In this fundamental task, AI significantly enhances the sensitivity of early cancer screening programs. For instance, emerging DL models, such as AI-guided colonoscopy systems, have increased adenoma detection rates by approximately 10%, while AI-assisted liquid biopsy models analyzing cfDNA, such as CancerSEEK (Cohen et al., 2018) or DELFI (Cristiano et al., 2019), are revolutionizing minimally-invasive early detection (Dellamonica et al., 2025). Beyond unimodal approaches, integrating multiple omics layers offers even greater diagnostic sensitivity. Driven by the clinical need to capture complex biological interactions, the landscape of ML tools tailored for multi-omics integration in cancer has rapidly expanded (Cai et al., 2022). For enabling early cancer detection, large-scale ML frameworks, such as iClusterPlus (Mo et al., 2013; Mo and Shen, 2026), iClusterBayes (Mo et al., 2018) and MOFA+ (Argelaguet et al., 2020), or DL frameworks, such as MOGONET (Wang et al., 2021) and CustOmics (Benkirane et al., 2023), have been developed for the integration of multi-omics, including epigenomic and transcriptomic data.

Multimodal ML applications have also come to aid in the field of radiology, primarily for disease classification, by integrating radiographic images with patient history and clinical notes, leading to substantially enhanced diagnostic accuracy (Haq et al., 2025). Foundational open-source infrastructures, such as MONAI (Cardoso et al., 2022), are able to integrate diverse medical imaging and clinical datasets for broad pan-cancer diagnostic tasks. For more targeted applications, custom pipelines such as RadGenNets (Tripathi et al., 2022) successfully integrate imaging and genomic data to assist in early detection.

DL has also become highly established in histopathology, where models are able to differentiate tumor tissue from healthy tissue on WSIs, using supervised learning paradigms (Unger and Kather, 2024). A wide variety of ML and DL algorithms, including Support Vector Machines (SVM), CNNs, bidirectional Recurrent Neural Networks (BiRNN), and Naive Bayes, have been deployed to improve breast cancer classification (Cruz-Roa et al., 2017; Chen D. et al., 2018, 2021; Wekesa and Kimwele, 2023; Renjith and Jose, 2023). More recent frameworks incorporate the Internet of Medical Things (IoMT) utilizing Gated Recurrent Units (GRU) and blockchain technology for secure detection and patient management (Wekesa and Kimwele, 2023; Aldhyani et al., 2023). To further maximize precision, multimodal deep CNN models have been trained on both digital images and electronic health records, effectively differentiating normal from abnormal breast tissues by extracting complex morphological, genetic, and binding site features (Wekesa and Kimwele, 2023; Renjith and Jose, 2023). Frameworks such as CONCH (Lu et al., 2024) and HONeYBEE (Tripathi et al., 2025) integrate WSIs with clinical and genomic data, utilizing cross-modal attention in order to achieve holistic diagnostic data fusion. Similarly, advanced pipelines, such as PathOmics (Ding et al., 2023) and PaGeFin (Monabbati et al., 2025), bridge subvisual morphological tissue features with genomic profiles and chromatin accessibility states for uncovering novel diagnostic signatures.

### 4.2 Tumor subtyping and grading

DL is able to solve complex subtyping and grading tasks in solid tumors. For example, weakly supervised learning models have been used for automated tumor segmentation and were able to accurately predict Gleason grades in prostate cancer (Ström et al., 2020; Unger and Kather, 2024). To further refine morphological grading across solid tumors, open-source frameworks, such as Porpoise (Chen et al., 2022b), as well as custom pipelines, such as Pathomic Fusion (Chen et al., 2022a), leverage attention mechanisms and graph convolutional networks to seamlessly integrate WSIs with genomic data. Currently, these advanced DL applications are being widely implemented for subtyping various types of malignancy, including breast cancer (Chen D. et al., 2021; Li et al., 2022), non-small-cell lung cancer (Li X. et al., 2025), glioblastoma (Huang et al., 2025), soft tissue tumors (Hofvander et al., 2026), or gastrointestinal cancers (Wang et al., 2023; Marouf et al., 2025).

In the context of breast cancer, the KDBTC model (Chen D. et al., 2021) focuses on subtyping by incorporating hierarchical attention-based bidirectional RNNs (HA-BiRNNs) and tree-structured GRUs to encode syntax and semantic information directly from multimodal clinical and imaging reports, while other tools, such as MoGCN (Li et al., 2022), combine GCN with AEs and similarity network fusion for precise multi-omics data integration and molecular subtyping in breast cancer. However, recent benchmarking efforts have highlighted the complementary strengths of statistical and DL approaches, and a comparative analysis in breast cancer subtype classification demonstrated that the ML tool MOFA+ outperformed the DL-based MoGCN in feature selection and clustering robustness, achieving a higher F1-score (0.75 vs. 0.70) and identifying a more extensive range of relevant biological pathways (121 vs. 100), most likely due to MoGCN’s focus on minimizing reconstruction error, which might result in less interpretable features (Omran et al., 2025).

In the field of onco-pneumology, DeepTFtyper (Li X. et al., 2025) proposes a model optimized specifically for NSCLC classification by mapping architectural tumor profiles from WSIs onto their corresponding proteomic signatures. In neuro-oncology, predicting tumor grading in gliomas relies heavily on multimodal approaches, especially as a consequence of the updated classification standards from the World Health Organization, which currently mandate the integration of molecular features as decision standards alongside traditional histopathological evidence. Therefore, custom radiogenomics ML pipelines that integrate MRI-based radiomic features with genomic data have been successfully used to predict oncogenic signaling pathways for the precise molecular characterization of glioblastomas (Huang et al., 2025). Expanding beyond common epithelial cancers, novel ML pipelines have been developed for subtyping soft tissue tumors, integrating transcriptomics, genomics, and clinical data to significantly refine tumor classification and identify prognostic transcriptomic signatures (Hofvander et al., 2026).

### 4.3 Prognosis and treatment response

Multimodal AI excels in advanced predictive tasks by anticipating the survival probability and therapeutic response. Early multimodal models successfully combined WSIs with genomic data to improve survival predictions across numerous cancer types, significantly surpassing traditional clinical biomarkers. Similarly, modern multimodal transformers, such as PathOmics (Ding et al., 2023), use unsupervised pretraining to connect morphological tissue features with genomic profiles, providing useful insights regarding survival outcome prediction. At a broader foundational level, the recently released HONeYBEE (Tripathi et al., 2025) utilizes cross-modal attention and adaptive fusion to integrate clinical, imaging, and genomic data, enabling personalized prognostic prediction.

Beyond imaging, multi-omics data is also used for precise cancer treatment, employing methods such as denoising AEs in order to establish robust representations for cancer-risk estimation (Chai et al., 2021; Wekesa and Kimwele, 2023). To scale these capabilities, frameworks like MOFA+ (Argelaguet et al., 2020) and MUON (Bredikhin et al., 2022) enable the integration of omics data from many layers, such as epigenomics, transcriptomics and proteomics, for estimating survival risk-scores and outcomes. Furthermore, exploring the tumor microenvironment at a single-cell resolution is crucial for anticipating therapeutic failures. Seurat v4 (Hao et al., 2021) is a pipeline able to integrate multimodal single-cell data to identify cellular biomarkers specifically associated with immunotherapy resistance. In the same regard, for therapy response prediction, MSDLM evaluates cellular patterns of anti-tumor immunity in colorectal cancer, outperforming standard clinical and molecular parameters (Wekesa and Kimwele, 2023; Foersch et al., 2023).

Such AI multimodal integrative tools have been successfully used and validated in many types of cancer. In head and neck oncology, SMuRF (Song et al., 2025) integrates CT imaging with multi-region WSIs for outcome prediction in HPV-associated oropharyngeal squamous cell carcinoma. Additionally, the GNPC (Zhou et al., 2025) framework utilizes GNN to integrate digital pathology with clinical information, providing precise risk stratification for distant metastasis and overall survival in nasopharyngeal carcinoma. In advanced NSCLC, integrating medical imaging, digitized programmed death-ligand 1 immunohistochemistry slides and genomic features successfully predicts critical immunotherapy responses (Vanguri et al., 2022; Unger and Kather, 2024). This strategy of combining morphology with molecular profiles is also highly effective in gastrointestinal malignancies; for example, the PSCRC (Lou et al., 2025) pipeline integrates digital pathology with transcriptomic data using multi-instance learning and interpretability modules to predict overall survival and adjuvant chemotherapy benefits in colorectal cancer. ViT (Ye et al., 2025) pipelines have been developed for estimating the prognosis of gastric cancer by combining immune-related RNA-seq signatures with subvisual WSI features. Before turning to their overall benefits, limitations, and future direction, the following section compares these frameworks across fusion strategy, architecture, interpretability, robustness, and clinical applicability.

## 5 Critical appraisal and future directions

### 5.1 Critical comparison of frameworks and fusion strategies

The frameworks summarized in Table 2 differ first in their fusion strategy, and each choice entails a distinct trade-off. Early, feature-level fusion is computationally economical and preserves the joint distribution of the modalities, but it presupposes reliable inter-modality alignment and scales poorly as dimensionality grows, which is why it is largely confined to the classical machine-learning pipelines in this review, such as the radiogenomic glioblastoma and soft-tissue transcriptomic pipelines (Marouf et al., 2025; Ahanger et al., 2025). Late fusion is the most robust to missing or heterogeneous modalities because each stream is modeled independently, yet this same independence discards the cross-modal interactions that frequently carry the strongest prognostic signal (Al-Zoghby et al., 2025). Intermediate, joint fusion, which dominates the recent deep-learning models in Table 2, offers the best balance by learning non-linear cross-modal representations before prediction, at the cost of greater data and computational requirements and reduced transparency (Guarrasi et al., 2025). Hybrid and adaptive schemes such as HONeYBEE extend this expressiveness further but add architectural complexity that can hinder reproducibility (Tripathi et al., 2025).

A second axis of comparison is the underlying architecture. Classical ML ensembles remain attractive for their low data and computational footprint and their intrinsic interpretability through feature-importance measures, but they capture cross-modal and non-linear structure less effectively than deep models (Ahanger et al., 2025). Graph-based methods suit the relational structure of omics networks and lend themselves to biomarker attribution, as in the multi-omic biomarker identification reported for MOGONET (Wang et al., 2021). Transformer-based models and attention-based designs (i.e., MCAT and Pathomic Fusion) are the most useful for modeling long-range cross-modal dependencies and currently define the state of the art for survival prediction, but they require large amounts of data, are computationally demanding, and offer only the limited and much-debated interpretability of attention maps (Chen R. J. et al., 2021; Chen et al., 2022a). Foundation models form the newest tier: pretraining on large corpora yields transferable representations that reduce task-specific training and can achieve strong zero-shot performance, as reported for CONCH, although at the price of very large pretraining data and compute and the least transparency of any class (Lu et al., 2024; Tripathi et al., 2025).

Across these families, three cross-cutting patterns emerge. First, while attention weights remain the primary mechanism for interpreting deep learning architectures, explicit explainability techniques are increasingly represented. As Table 2 shows, alongside the SHAP- and Grad-CAM-based colorectal pathomics signature (Lou et al., 2025), explicit attribution frameworks like Integrated Gradients, Grad-CAM, and SHAP are now incorporated into broad open-source ecosystems such as MONAI (Cardoso et al., 2022) and task-specific pipelines including Porpoise (Chen et al., 2022b), SMuRF (Song et al., 2025), and UMPSNet (Zhang et al., 2025). Emerging explainability approaches, such as counterfactual explanations (Ji et al., 2025) and specialized morphology-aware graph architectures (Li X. et al., 2025), are also gaining traction. Beyond the attribution and attention methods already deployed, several widely adopted XAI techniques, including LIME, saliency maps, uncertainty estimation, and concept-based explanations, remain largely unexploited in the multimodal oncology models surveyed here and represent a clear opportunity to deepen interpretability (Wang et al., 2026). Across these approaches, explainability is increasingly consequential not only for interpreting how heterogeneous modalities drive a prediction, but also for establishing clinician trust, meeting the regulatory-acceptance requirements discussed in Section 5.2, and detecting the demographic biases noted there.

Second, robustness and generalizability remain severely constrained by an overwhelming reliance on retrospective, single-institution cohorts such as TCGA. External or prospective validation is reported for only a fraction of cancer-specific pipelines, such as in oropharyngeal carcinoma (Song et al., 2025). External validation in CONCH (a foundation model; Lu et al., 2024) and in UMPSNet (Zhang et al., 2025), which reports zero-shot external validation, represents an encouraging shift. Finally, greater architectural complexity does not guarantee superior performance, since large-scale benchmarking demonstrates that fusing additional modalities can sometimes degrade rather than improve accuracy (Li et al., 2024). These observations, together with the widespread and noticeable lack of standardized performance metrics across the “Reported performance” column of Table 2, directly motivate the benefits and limitations discussed below.

### 5.2 Benefits and limitations

The clinical implementation of personalized medicine has historically been constrained by a few biomarkers capable of explaining patient outcomes. However, AI models currently demonstrate exceptional performance in predicting clinical outcomes, including survival probability, treatment response, tumor recurrence, and radiation toxicity, utilizing both unimodal and multimodal data architectures (Lipkova et al., 2022). On one hand, multimodal integration has been shown to significantly enhance diagnostic precision, with reported performance improvements typically ranging from 5 to 15% over conventional unimodal baselines (Jandoubi and Akhloufi, 2025). On the other hand, the assumption that maximal data integration always yields superior outcomes has been recently challenged and needs to be addressed with a critical eye. Large-scale benchmarking studies, such as the one conducted by Li et al. (2024), concluded that amalgamating numerous omics types can actually impede performance, with models often achieving peak accuracy using only a handful of highly informative data modalities (ex. only using methylation data instead of combining five omics layers). Furthermore, the emergence of generalist models such as ChatGPT-4 V has introduced a new layer of inconsistency due to performance variability across different types of cancer and, in certain scenarios, have been shown to underperform compared to their unimodal, text-only versions (Al-Zoghby et al., 2025). The broader oncological applications of such LLMs are introduced in Section 3.

Despite the significant performance enhancements shown by AI multimodal integration tools, critical evaluation reveals substantial limitations that span five interdependent dimensions: clinical validation, model explainability, data quality, algorithmic bias, and clinical implementation (Jandoubi and Akhloufi, 2025). These five dimensions are examined below. Integrative analysis in precision oncology is fundamentally challenged by the diversity of multi-omics data formats, which frequently require models able to simultaneously reconcile binary mutation indicators, continuous microarray measurements, and discrete RNA-seq count data. Due to the fact that these disparate data types possess unique sources of biological and technical variation, they necessitate specialized modeling strategies that often exceed the flexibility and computational efficiency of current analytical frameworks (Mo et al., 2018). Another challenge in clinical translation is the lack of standardized fusion pipelines, which exacerbates data fragmentation, leads to inconsistent reporting, and severely limits the reproducibility of results across independent studies. With regard to validation, the current evidence base remains fragile; research relies heavily on retrospective cohorts, such as TCGA, with a notable scarcity of prospective, multi-center validation required to ensure broad generalizability. This dependence on retrospective, single-institution data introduces a substantial risk of overfitting and information leakage, and is compounded by inconsistent reporting, which the adoption of AI-specific reporting standards, including the TRIPOD+AI statement and related guidelines such as CLAIM and DECIDE-AI, would help to remedy (Collins et al., 2024). Technical barriers also persist, including the extreme computational demands required for high-dimensional data integration and the prevalent underutilization of biologically informed AI. This underutilization prioritizes raw performance over the explanatory power which is required for medical trust (Huang et al., 2020; Wekesa and Kimwele, 2023). Regarding explainability, most deployed multimodal architectures still operate as opaque “black-box” systems that obscure how individual modalities contribute to a given prediction, which undermines clinician trust, complicates error auditing, and impedes regulatory acceptance.

In addition to technical and translational challenges, comprehensive data collection constitutes the primary bottleneck in training DL solutions for precision oncology. While histopathological data is routinely acquired, extensive genomic cohorts are costly and difficult to establish, limiting the rapid development of multi-omic approaches (Unger and Kather, 2024). In terms of data quality, the intrinsic reliability of available medical data is often compromised by modality-specific noise, mismatched samples, and the strict necessity for extensive expert annotation. A distinct concern is algorithmic bias: numerous datasets frequently exhibit demographic skew based on ethnicity, sex, socio-economic circumstances, or institutional origin, which directly translates into biased model behavior and systemic inequality (Wekesa and Kimwele, 2023; Unger and Kather, 2024; Jandoubi and Akhloufi, 2025). Furthermore, the pervasive issue of missing data severely impacts model training, although advanced techniques such as synthetic data generation, up-sampling, and attention mechanisms are actively being implemented to mitigate these constraints (Lipkova et al., 2022; Wekesa and Kimwele, 2023; Waqas et al., 2024).

Finally, clinical implementation raises its own set of barriers. The deployment of multimodal deep learning models in healthcare introduces profound legal and ethical challenges regarding privacy and data security, directly reflected at the patient level, which tend to show increasingly mixed sentiments towards the idea of having reduced personal interactions with medical providers (Kaushik and Rapaka, 2024). To mitigate the risks of discrimination and ensure robust data protection without compromising continuous scientific progress, Federated Learning (FL) has emerged as a critical architectural approach. This paradigm enables the localized training of large multimodal models across multiple independent institutions, safeguarding patient privacy by completely eliminating the need for direct data sharing (Wekesa and Kimwele, 2023; Waqas et al., 2024).

Beyond these technical and ethical barriers, the regulatory pathway for multimodal AI remains a decisive obstacle to clinical adoption, as the overwhelming majority of published models are hypothesis-generating prototypes that have not undergone the prospective validation required for regulatory clearance (Unger and Kather, 2024). Most such tools fall under the regulatory category of Software as a Medical Device, a framework originally designed for static algorithms that is difficult to reconcile with models intended to learn and adapt over time. To address this tension, the United States Food and Drug Administration has issued Good Machine Learning Practice guiding principles and now permits manufacturers to specify a Predetermined Change Control Plan, which pre-authorizes a defined envelope of future model updates without a new submission (US Food and Drug Administration, 2025). In the European Union, the recently adopted AI Act classifies medical AI as high-risk and mandates rigorous oversight, yet substantial gaps persist regarding risk categorization, post-market monitoring, liability allocation, and consistent implementation across member states (Vardas et al., 2025). For multimodal systems specifically, regulators have no standardized validation route for tools that fuse heterogeneous data streams, and demonstrable explainability together with prospective, multi-center evidence is increasingly treated as a precondition for approval, reinforcing the reporting and validation standards discussed above. Finally, data-protection regimes such as the GDPR and HIPAA constrain the cross-institutional data sharing on which multimodal training depends, further motivating privacy-preserving strategies such as federated learning (Kaushik and Rapaka, 2024).

### 5.3 Future directions

Realizing the full clinical potential of multimodal AI will depend on addressing the gaps identified above. Immediate priorities include large-scale prospective and external, multi-center validation, the adoption of standardized performance metrics and reporting to enable fair comparison across models, and the wider integration of explainability techniques in order to strengthen clinician trust and support regulatory acceptance. Beyond these consolidations, several emerging paradigms are poised to reshape the field. Generalist foundation models are expected to provide increasingly transferable representations across cancer types, whereas Retrieval-Augmented Generation (RAG) frameworks and AI agents offer a promising route to couple multimodal patient data with continuously updated biomedical knowledge bases, thereby enabling more reliable, explainable, and context-aware clinical decision support in precision oncology.

## 6 Conclusion

In conclusion, the rapid technical advancements achieved over recent years have established DL models as increasingly powerful, robust, and generalizable tools within clinical oncology. Multimodal DL now stands at the forefront of a profound healthcare paradigm shift, successfully driving the integration of diverse, high-dimensional datasets, spanning complex multi-omics profiles, digitized histopathology, and macroscopic radiological imaging. This unprecedented data convergence facilitates a far more thorough, holistic, and nuanced comprehension of patient-specific health and underlying tumor biology. By seamlessly streamlining complex data processing and bridging the gap between genotype and phenotype, multimodal artificial intelligence significantly enhances diagnostic precision and predictive accuracy. However, the current literature highlights the necessity of moving toward task-specific, biologically-informed architectures that prioritize the quality of modality alignment over the quantity of integrated data. Realizing this potential will further require rigorous external and prospective validation, greater model explainability, and clearer regulatory pathways, with emerging foundation models and retrieval-augmented, agent-based systems representing the most promising next steps. Ultimately, continuing to refine these integrated systems and overcoming current translational data challenges will unlock their full clinical potential, thereby paving the way for the widespread implementation of truly personalized, data-driven precision medicine.
